# Conversion of a Polyolefin Mixture into Light Olefins by Fast Pyrolysis and In-Line Catalytic Cracking on HZSM‑5

**DOI:** 10.1021/acs.energyfuels.5c06791

**Published:** 2026-04-20

**Authors:** Biagio Ciccone, Katrin Santin, Gartzen Lopez, Maite Artetxe, Maider Amutio, Martin Olazar, Massimo Urciuolo, Giovanna Ruoppolo

**Affiliations:** † Department of Applied Science and Technology, 19032 Politecnico di Torino, C.so Duca degli Abruzzi 24, Torino 10129, Italy; ‡ Istituto di Scienze e Tecnologie per l’Energia e la Mobilità Sostenibili, 9327 Consiglio Nazionale delle Ricerche, P. le V. Tecchio 80, Napoli 80125, Italy; § Department of Chemical Engineering, 200145 University of the Basque Country (EHU), P.O. Box 644, Bilbao E48080, Spain; ∥ IKERBASQUE, Basque Foundation for Science, Bilbao 48009, Spain

## Abstract

A model polyolefin mixture—composed of 50% HDPE, 40% LDPE, and 10% PP—has been converted into valuable light olefins on an HZSM-5-based catalyst in a two-step continuous process: fast pyrolysis in a conical spouted bed reactor followed by upgrading in a catalytic fixed-bed reactor. The effect of the operating conditions in the catalytic step (space-time of 10 and 15 g_catalyst_ min g_plastic_^–1^ and temperature of 450 and 500 °C) on product yields and catalyst stability has been investigated up to a time on stream (TOS) of 4 h. Light olefins represented the most abundant product, with a maximum yield of 77% obtained at 500 °C with a space-time of 10 g_catalyst_ min g_plastic_^–1^ under zero-time conditions. Further increase in space-time led to a reduction in light olefin yield and to an increase of benzene, toluene, and xylene isomers (BTX) as well as light alkanes. Characterization of spent catalyst samples (coke deposition, physical properties) revealed the presence of two different types of coke and a higher coke deposition in the first section of the catalytic bed (in contact with incoming waxes). The catalyst proved to be stable at 500 °C, while higher deactivation was observed after 4 h of operation at the lower temperature (450 °C).

## Graphical abstract

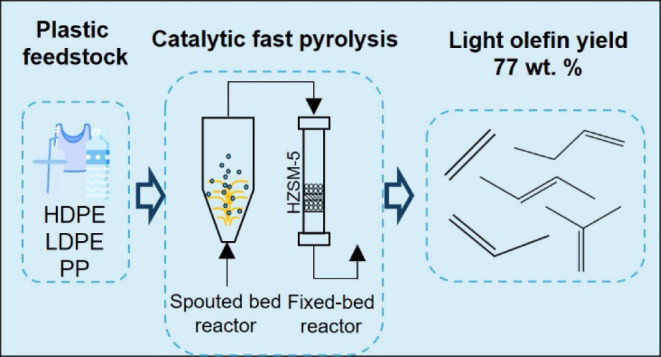

## 1 Introduction

Global plastic production reached 430.9 million tons in 2024, yet only 10.3% was mechanically recycled, biobased, or chemically recycled.^1^ The accumulation of plastic waste poses severe environmental challenges, making efficient waste management and valorization strategies essential to achieve circularity in the plastics sector. Among thermochemical conversion technologies, pyrolysis offers a versatile route to convert carbon-rich materials into energy carriers and/or valuable chemical feedstocks, depending on the process configuration and downstream integration.^2–5^

However, conventional thermal pyrolysis occurs via random scission of polymeric chains and typically yields low-quality products due to its poor selectivity.^2,6^ To overcome this limitation, catalytic upgrading is used to direct the process toward target products, such as light olefins, monoaromatics, and hydrocarbons in gasoline or diesel range.^7–10^ Among the catalysts investigated in the literature, zeolites (particularly HZSM-5, HY, and Hβ) are the most commonly employed for the catalytic cracking of plastic waste.^11,12^ Catalyst properties—particularly pore structure and acidity—strongly influence the reaction network of the upgrading process (homolytic scissions, alkylation, aromatization, isomerization), thus shaping the final product distribution.^13,14^ While large pore size and moderate acidity—as in the case of HY and Hβ—lead to a higher production of liquid hydrocarbons in the gasoline or diesel range,^15–17^ HZSM-5 has drawn attention due to its ability to selectively promote the formation of light olefins and, to a certain extent, aromatic hydrocarbons.^18–20^ The shape-selective structure of HZSM-5 limits the nucleation of polyaromatic hydrocarbons, increasing the production of light olefins and mitigating deactivation.^21–25^

Light olefins are key intermediates for petrochemical synthesis, and their selective recovery from plastic waste represents a promising route for chemical recycling.^9,12^ Polyolefins are ubiquitous in packaging (36% share of global market size in 2024), construction, and consumer goods. Their relatively safer and cleaner thermal degradation, compared to heteroatomic plastics (e.g., polyethylene terephthalate, polyvinyl chloride), makes polyolefin valorization via catalytic pyrolysis a promising opportunity for a decentralized waste-to-resource process. The suitability of conical spouted-bed reactors (CSBR) for continuous catalytic pyrolysis of polyolefins is well documented in the literature for both in situ and ex situ configurations.^22,26–30^ The cyclic movement of bed particles guarantees uniform plastic melting, sand particles coating,^31^ and high heat and mass transfer rates (fast devolatilization), preventing defluidization issues typical of fluidized-bed reactors.^32^ Compared to in situ processes, the ex situ configuration decouples plastic pyrolysis from catalytic cracking, allowing precise control of temperature and space-time, thus improving product quality and selectivity toward selected products, i.e., light olefins.^30,33^

In this work, we investigate the influence of space-time and cracking temperature on the activity and stability of a highly acidic HZSM-5 catalyst during in-line upgrading of pyrolysis volatiles derived from a model polyolefin mixture (HDPE, LDPE, and PP). The use of a model mixed polyolefin feed allows the investigation of potential synergistic effects and component interactions during plastic degradation and upgrading, which may lead to pyrolysis behavior different from that of pure HDPE. More in detail, the effect of catalyst space-time (i.e., mass of catalyst per unit feed mass flow rate) is examined under fresh-catalyst conditions in the range 8–20 g_catalyst_ min g_plastic_^–1^, while the evolution of product yields and catalyst deactivation over time is analyzed at varying temperatures (450 and 500 °C) and space-times (10 and 15 g_catalyst_ min g _plastic_^–1^). Coke deposition and the degradation of zeolite properties are monitored along the reactor bed, divided into two sections, catalytic beds 1 (CB1) and 2 (CB2), to elucidate the axial evolution of spent catalyst properties. A deeper understanding of these parameters is essential to design robust catalytic processes for circular plastic-to-chemicals technologies. The findings contribute to optimizing catalytic pyrolysis schemes for light-olefin production and advancing circular plastic-to-resource conversion.

## 2 Experimental Section

### 2.1 Feedstock and Catalysts

A polyolefin mixture consisting of 50% HDPE, 40% LDPE, and 10% PP was selected as a simplified model system to move beyond single-polymer experiments and to explore potential interactions among different polyolefins while maintaining a controlled and reproducible feed composition. The polymers were provided by Dow Chemical (Tarragona, Spain) in the form of chippings of 4 mm, and their main properties are reported in Table 1. Ultimate analysis was performed using a LECO CHN 828 elemental analyzer and reflects the theoretical composition of the plastics under examination. The higher heating value (HHV) was measured by differential scanning calorimetry (Setaram TG-DSC-111) and an isoperibolic bomb calorimeter (Parr 1356).

**Table 1:** Characteristics of the Polyolefins Used in This Work

	HDPE	LDPE	PP
C (wt %)	85.19	85.69	85.47
H (wt %)	14.81	14.31	14.53
Molecular Weight (kg mol^–1^)	46.2	92.2	50–90
Polydispersity	2.89	5.13	2.0
HHV (MJ kg^–1^)	43	43	44

A commercial catalyst based on HZSM-5 zeolite and γ-Al_2_O_3_ as a binder (reference code HSZ-840HOD1A, Si/Al = 40) was supplied by TOSOH Chemicals (Tokyo, Japan) and has been used to conduct experiments. The catalyst was supplied in the form of white pellets (1.5 mm diameter) and was sieved in the 1.0–1.8 mm range.

The catalyst in its acidic form has been pretreated in air at 550 °C for 2 h to remove moisture and eventually adsorbed compounds. The physical properties of the catalyst (e.g., BET surface area, pore volume, and pore diameter) were measured by using a Micromeritics ASAP 2010 analyzer. The catalyst has an equal amount of microporous—from the zeolite itself—and mesoporous pore volume (Table 2), thanks to the presence of γ-Al_2_O_3_. The presence of a mesoporous structure is a recognized factor for the attenuation of coke deposition in the pore structure of the zeolite.^21,34,35^ The acidic properties of the catalyst were characterized by the temperature-programmed desorption of NH_3_ (TPD-NH_3_) using a Micromeritics AutoChem II chemisorption analyzer. The acidity distribution has been calculated from the relative area of the peaks obtained from Gaussian fitting of the TPD curve according to the following classification: weak acidic sites (<150 °C), average acidic sites (150–350 °C), and strong acidic sites (>350 °C).^36^ Results revealed that the catalyst is highly acidic, with a non-negligible share of strong acid sites (Table 2). The nature of the acid sites (Brønsted and Lewis) of solid samples was analyzed by FTIR (Fourier transform infrared spectroscopy) of adsorbed pyridine on a Nicolet 6700 FTIR spectrometer using a Specac high temperature/high pressure (HTHP).

**Table 2:** Fresh Catalyst Properties

BET surface area (m^2^g^–1^)	402
Micropore area (m^2^ g^–1^)	152.3
Micropore volume (cm^3^ g^–1^)	0.14
Mesopore volume (cm^3^ g^–1^)	0.14
Average pore diameter (Å)	75
Total acidity $mmol_{NH_{3}}\;g^{‐1}$	1.58
Acidity distribution by TPD (%) (Weak/Medium/Strong)	29.4/49.7/20.9
Brønsted/Lewis sites ratio	6.33

Spent catalyst samples have also been characterized in terms of physical properties (BET surface area) and coke deposition. Coke deposition was quantified by temperature-programmed oxidation (TPO) using a thermobalance (T.A. Instruments TGA Q5000). The protocol used is as follows: (i) sample heating under N_2_ atmosphere up to 200 °C for 20 min to remove chemisorbed species and moisture; (ii) cooling to 100 °C and switching to air; (iii) heating to 800 °C at 5 °C min^–1^ for 10 min for complete coke combustion.

### 2.2 Experimental Equipment and Procedure

A schematic representation of the two-step catalytic pyrolysis plant used in this study is reported in Figure 1. The solid feeding system consists of a cylindrical vessel (30 mm ID) equipped with a vertical shaft and a piston. A vibration module provides the necessary mobility to ease the solid particles to fall into the feeding tube, while the piston slowly rises to the top. The feeding tube is connected vertically to the spouted bed reactor and is refrigerated by a tap water cooling jacket to prevent premature plastic melting. Moreover, a nitrogen stream is supplied to the top of the feeder to avoid the rise of pyrolysis volatiles from the reactor. The speed of the piston can be regulated to obtain a solid feed between 0 and 5 g min^–1^.

**Figure 1:**
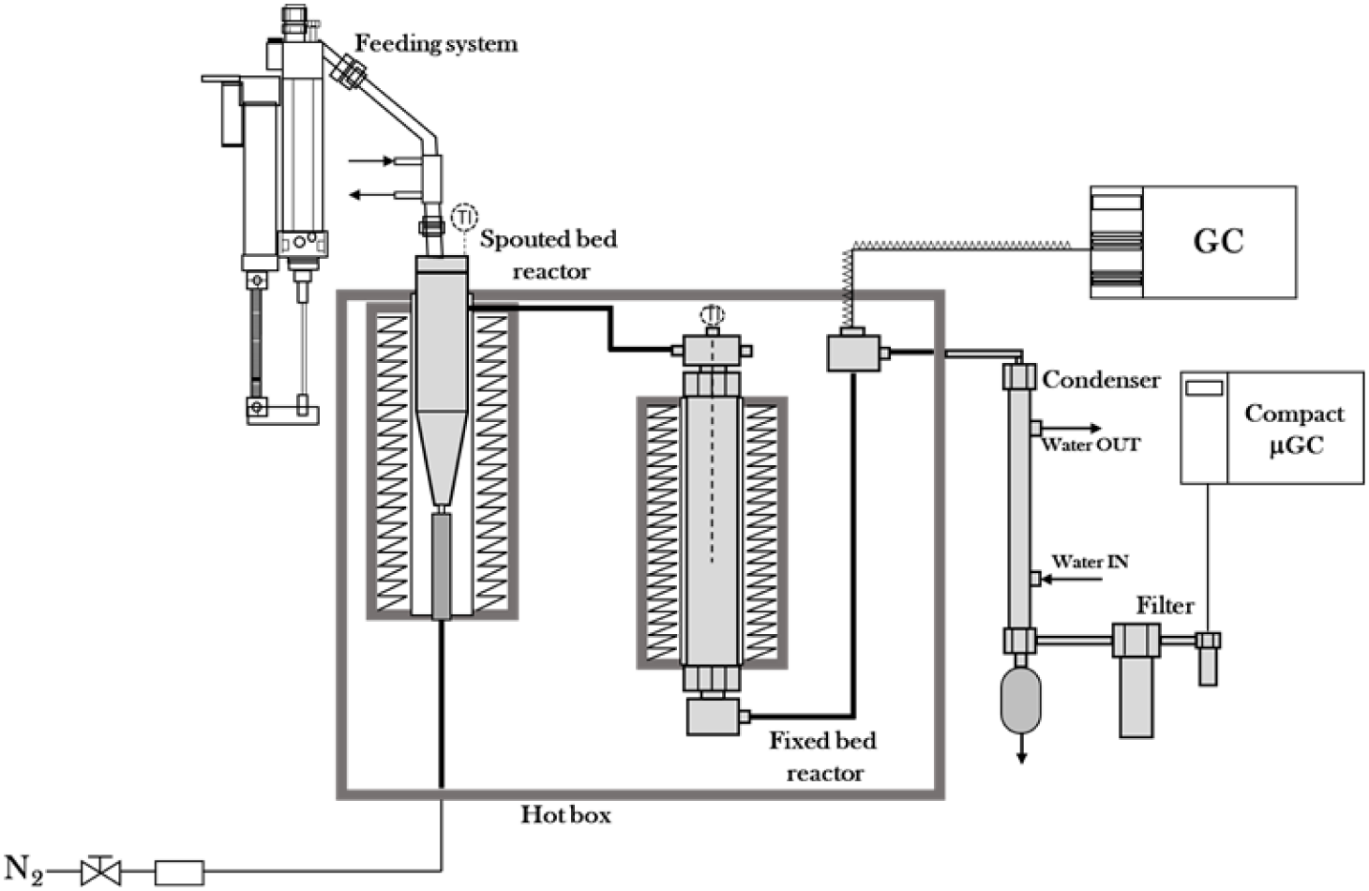
Schematization of the bench-scale plant used for pyrolysis and in-line catalytic cracking of polyolefins.

The conical spouted-bed reactor is characterized by a combination of a conical section (at the bottom) and a cylindrical section. The main dimensions are as follows: the total height of the reactor is 297 mm; the height of the conical section is 77 mm, and its tilt angle is γ=30°. The diameter of the cylindrical section of the reactor is 54.8 mm; the diameter of the base of the conical section is 12.5 mm; and the gas nozzle diameter is 4.9 mm. Moreover, the reactor is provided with a nonporous draft tube which allows for improving bed stability and reducing gas flow rate.^37–39^ The detailed design of the draft tube can be found elsewhere.^40^ A shell oven (1500 W) ensured reactor heating, and a K thermocouple was used to monitor the temperature of the reactor. The operating conditions adopted during the experimental campaign guaranteed a stable spouting regime during the experimental campaign.

The volatiles produced during plastic pyrolysis are conveyed to the in-line catalytic fixed-bed reactor. Both reactors are located inside a stainless-steel forced convection oven (1250 W) kept at 300 °C to prevent the condensation of heavy pyrolysis volatiles in the tubes after the pyrolysis reactor. The catalytic reactor consists of a stainless steel tube (440 mm long, 38.1 mm ID) equipped with a thermocouple to monitor the real temperature in the catalyst bed and a ceramic shell oven (550 W).

A constant feed of 1 g min^–1^ was used in this study. The temperature of the pyrolysis reactor was fixed at 500 °C during the tests based on previous experience in plastic pyrolysis in the CSBR.^22,27,28,30,41,42^ The bed of the CSBR consisted of 80 g of sand (0.8–1.0 mm), and nitrogen was used to fluidize the material with a flow rate of 10 L min^–1^ at ambient conditions—corresponding roughly to 2 times the minimum spouting velocity. The attained conditions promote vigorous fluidization and ensure high heat and mass transfer, isothermicity of the bed, and avoid operational problems such as bed defluidization.

The influence of catalyst space-time at zero time on stream was studied between 8 and 20 g_catalyst_ min g_plastic_^–1^ at 500 °C. The volume of the catalytic bed was always 30 mL, and the mass of the catalyst was modified to obtain different values of space-time. The remaining volume was filled with inert coarse sand (1.2–2.0 mm). Moreover, the effect of operating conditions, space-time of 10 and 15 g_catalyst_ min g_plastic_^–1^, and cracking temperatures of 450 and 500 °C on catalyst stability was evaluated in long continuous runs. The evolution of product distribution with time on stream (TOS) was elucidated by conducting continuous experiments up to 240 min. Moreover, spent catalyst samples were collected at different TOS (after 60, 120, and 240 min) to monitor the degradation of catalyst features throughout the reaction. In these experiments, catalyst bed was divided into two distinct sections of equal mass by interposing a steel wire mesh to give insights into the axial distribution of coke deposition and catalyst’s properties deterioration.

A sample of the volatile stream from the fixed-bed reactor is subsequently sent to the in-line GC for analysis through a thermostated line (kept at 280 °C to avoid the condensation of heavy products), while the rest of the product stream is sent to the condensation system, consisting of a tap water-cooled hairpin condenser followed by a sintered steel filter and a coalescence filter for the complete condensation of volatile hydrocarbons. Quantitative characterization of the volatile stream was performed using an Agilent 8890 Gas Chromatograph equipped with an HP-PONA column and a flame ionization (FID) detector, which provides a proportional relationship between peak area and mass for hydrocarbons.^43^ The temperature program used in the chromatographic oven includes 2 min at 40 °C, followed by a ramp of 15 °C min^–1^ up to 320 °C and a 3 min isotherm at 320 °C. Permanent gas samples were collected downstream of the condensation system in Tedlar gas sampling bags and analyzed in a G.A.S. Compact GC^4.0^ chromatograph, equipped with two types of detectors (FID and thermal conductivity detector (TCD)) and four types of columns (Rtx-1, Rt-Q-Bond, Molecular Sieve 5A, MXT-Q Bond) arranged in three channels. Each measurement has been repeated at least twice to assess the reproducibility of the results, and differences were always below 5%.

## 3 Results and Discussion

### 3.1 Effect of Space-Time with Fresh Catalyst

The thermal fast pyrolysis of polyolefins at 500 °C in the spouted bed reactor generates a stream predominantly composed of waxes and heavy C_12_–C_20_ hydrocarbons (>90 wt %), with minor differences in the product distribution. Results from the pure thermal pyrolysis of HDPE, LDPE, and PP have been reported in Table 3.

**Table 3:** Composition of the Volatile Stream Produced during Thermal Pyrolysis of Pure Polyolefins in the CSBR

	HDPE		PP		LDPE	
Gas	1.5		1.3		1.7	
	Alkanes	0.4	Alkanes	0.4	Alkanes	0.5
	Alkenes	1.1	Alkenes	0.9	Alkenes	1.1
Oil	31.5		23.9		34.1	
	C_5_–C_11_	5.9	C_5_–C_11_	4.7	C_5_–C_11_	9.0
	C_12_–C_20_	25.6	C_12_–C_20_	19.2	C_12_–C_20_	25.1
Solid	67.0		74.8		64.2	
	Light waxes	29.5	Light waxes	32.5	Light waxes	31.5
	Heavy waxes	37.5	Heavy waxes	42.3	Heavy waxes	32.7

Moreover, the yield of the gasoline fraction (C_5_–C_11_) and especially gaseous hydrocarbons (C_1_–C_4_) is low.^6,30^ This result is associated with the combination of a high heating rate and low residence time of pyrolysis volatiles, which is typical of spouted bed reactors. It is to note that a similar product distribution was reported in previous literature on polyolefins fast pyrolysis.^44,45^ As previously reported, the mechanism can be schematized into chain initiation, propagation, and termination steps.^46^ During initiation, thermal energy induces homolytic C–C bond cleavage, forming unstable primary radicals that quickly convert to more stable secondary radicals. Primary radicals undergo β-scission to yield shorter radicals and ethylene, while secondary radicals produce alkenes and shorter chains during propagation. In termination, radical recombination forms *n*-alkanes and iso-alkanes.^7^ Subsequent isomerization and cyclization reactions generate cyclic hydrocarbons and aromatics. Thanks to the low residence time and fast heating of the polymer within the bed, the spouted-bed reactor guarantees a limited extent of β-scission and secondary reactions of isomerization and cyclization; thus, the prevailing products are waxes and heavy hydrocarbons. Moreover, under these conditions, no solid residue was obtained in the spouted bed reactor.

The influence of space-time on catalytic cracking of pyrolysis volatiles was evaluated during the first 5–10 min of steady-state operation to evaluate the performance of the fresh catalyst. Figure 2 shows the effect of space-time on product mass yields at 500 °C. Product fractions were grouped into different lumps: light olefins (C_2_–C_4_), light alkanes (C_1_–C_4_), BTX aromatics, other gasoline-fraction hydrocarbons (C_5_–C_11_, excluding BTX), and diesel fraction (C_12_–C_20_ hydrocarbons).

**Figure 2:**
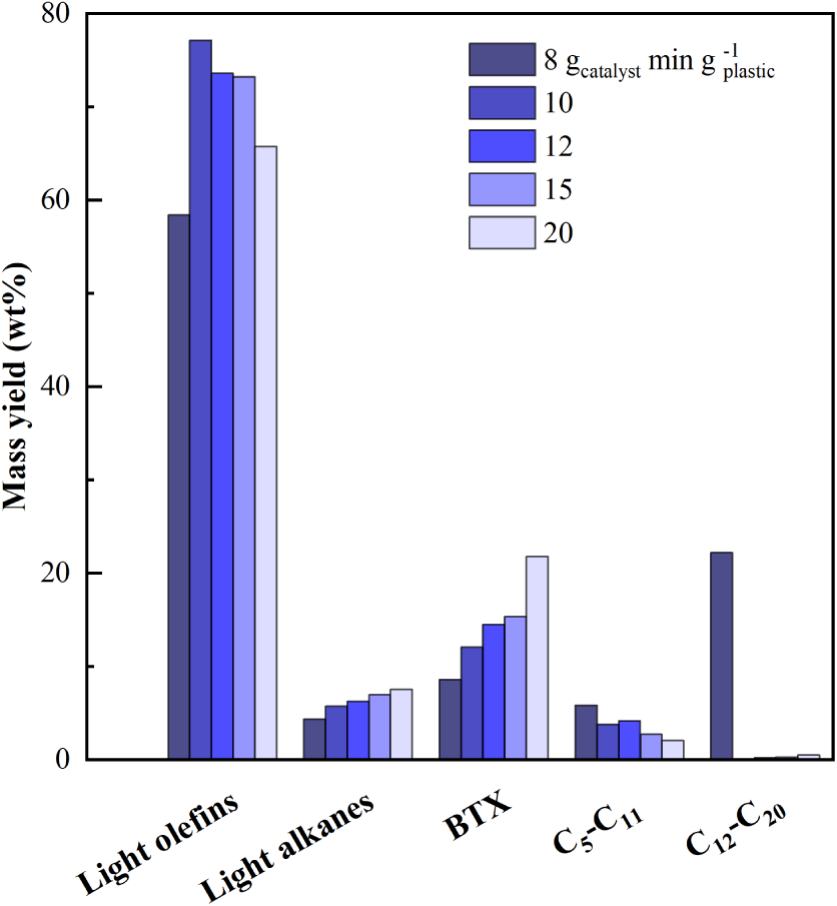
Effect of catalyst space-time on product fraction distribution (fresh catalyst) at 500 °C.

Increasing space-time from 8 to 20 g_catalyst_ min g_plastic_^–1^ enhanced cracking reactions, i.e., decreased gasoline and diesel fractions, while light alkanes increased from 4.3 to 7.5 wt % and BTX from 8.6 to 21.8 wt %. It is to be noted that even the operation with the lower space-time (8 g_catalyst_ min g_plastic_^–1^) studied allowed for the complete cracking of waxes (C_20_^+^ fraction) to lighter products. Moreover, a significant yield of diesel range (C_12_–C_20_) hydrocarbons was only observed at the lowest space-time (diesel fraction 22.2 wt.%), while complete conversion was obtained for τ ≥ 10 g_catalyst_ min g_plastic_^–1^.

The yield of light olefins followed a nonmonotonic trend peaking at 77 wt % at τ = 10 g_catalyst_ min g_plastic_^–1^ before decreasing to 65.7 wt % at τ = 20 g_catalyst_ min g_plastic_^–1^. This behavior confirms that it is necessary to strike a good balance between operating with enough catalyst to promote complete cracking of heavy hydrocarbons toward light olefins and avoiding too high catalysts space-times that promote a further conversion of light olefins by means of different secondary reactions. It is generally recognized that catalytic cracking of polyolefins over acidic zeolites begins with the protonation of the polymer chain at a Brønsted site, forming an unstable carbonium ion that rapidly decomposes into alkanes and more stable carbenium ions. Carbenium ions, stabilized by hyperconjugation and inductive effects—isomerize to secondary and tertiary forms, promoting further cracking.^47^ Rearrangement to tertiary carbocations increases product branching compared with thermal pyrolysis.^48^ The main propagation step is β-scission, where cleavage of the C–C bond β to the positively charged carbon generates a lighter olefin and a new carbenium ion. Secondary reactions—including isomerization, Diels–Alder cycloaddition, cyclization, dehydrogenation, and oligomerization—further expand the product distribution in the process. It is to note that this effect has not been previously observed by Artetxe and coworkers^30^ because of the lower range of space-time adopted (0 to 8 g_catalyst_ min g_plastic_^–1^). The lowest space-time used in the present study yielded 58.4 wt % of light olefins, consistent with results by Artetxe and coworkers for HDPE,^30^ suggesting that the behavior of mixed polyolefins under pyrolysis conditions are similar to that of pure HDPE. These results are aligned with those reported in previous literature and confirm that HZSM-5 efficiently cracks polyolefin-derived volatiles and selectively produces light olefins at relatively low temperature.^20,49–52^ Recent advancements in reactor design and catalyst modification have pushed the limits of light olefin production from polyolefins. For instance, Ngu and coworkers^53^ and Selvam and coworkers^54^ reported light olefin yields exceeding 80% using a single-stage, Joule-heated Reactive Pulse Heating (RPH) system, which benefits from extremely short contact times and precise temperature control. Similarly, Eschenbacher et al.^55^ achieved 79% yield from a synthetic polyolefin mixture using a single-shot tandem micropyrolyzer with a steamed P-doped HZSM-5 catalyst at 600 °C with a catalyst/feed ratio of 80. In another work,^50^ they obtained lower yields (69.3%) from LDPE using steamed HZSM-5 additives at 700 °C with a catalyst/feed ratio of 150. Bi and coworkers^200^ also reported a combined ethylene-propylene yield of 79% using a two-stage quartz-tube reactor with a dual zeolite system (LSP-Z100 + P-HZSM-5) at a cracking temperature of 540 °C. Netsch and coworkers^56^ reported a lower yield (56%) in an in situ auger reactor during pyrolysis (450 °C) of polyolefin waste. The results of this work are in agreement with recent advancements in the field and suggest that high olefin yield can be obtained at moderate temperature and low space-time without specialized reactor configurations in a continuous, scalable system. The production of BTX monoaromatics at expenses of light olefins is promoted by increasing space-time through secondary oligomerization and aromatization reactions, e.g., Diels–Alder cycloaddition of conjugated dienes, formation and aromatization of cyclohexanes or “hydrocarbon pool” mechanism within the pores of the zeolite.^7,57^ Since the hydrogen yield is very low (Figure 3), direct dehydrogenation is not the dominant aromatization reaction. Strong acidic sites are known to promote secondary aromatization reactions,^58^ and therefore the strong acidity of the zeolite explains the decrease of light olefins yield for high contact times and the relatively high BTX yield obtained in the process.^30,49,59^ The yield of individual BTX consistently increased with space-time, with toluene being the most abundant, followed by benzene and xylene isomers. More specifically, increasing the space-time from 8 to 20 g_catalyst_ min g_plastic_^–1^ resulted in an increase in toluene yield from 4.11 to 9.97%, while benzene and xylene yields increased from 2.37 to 7.62% and from 2.32 to 4.17%, respectively.

**Figure 3:**
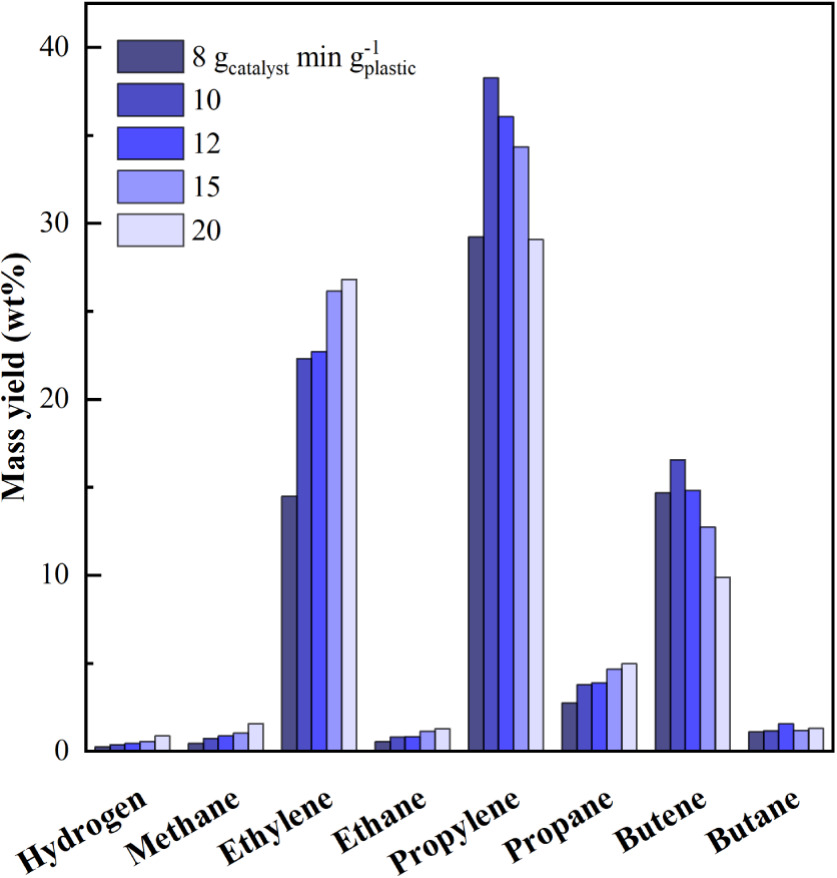
Effect of space-time on yields of individual gas-phase compounds.

The effect of space-time on the distribution of individual gaseous compounds is illustrated in Figure 3. Hydrogen is the least abundant compound in the gas phase, with yields ranging from 0.24 to 0.86 wt % with increasing space-time. The yield of single light alkanes (methane, ethane, propane, and butane) increases with space-time, while light olefins—except for ethylene—exhibit a nonmonotonic trend with a maximum (38.25 and 16.56 wt % for propylene and butenes, respectively). The yield of light alkanes is low because of the low residence time in the reactor, which reduced H-transfer reactions and bimolecular cracking to methane and ethane. Ethylene concentration steadily increased from 14.48 wt % to 26.80 wt % with increasing space-time, as it is a reaction end product, less subject to secondary transformations than olefins with a higher molecular weight (C_3_–C_4_). This behavior was observed in the literature.^49,60^ As a consequence of increasing secondary reactions, the relative proportions of ethylene, propylene, and butenes also change with space-time. Specifically, the propylene/ethylene ratio decreases (from ∼2.02 to ∼1.08), while the propylene/butenes and ethylene/butene ratios increase (∼1.99→∼2.94 and ∼0.98→∼2.71). The gas phase is richer in C_3_–C_4_ than C_1_–C_2_ and contains a higher proportion of C_3_ than C_4_ compounds, as observed elsewhere in the catalytic pyrolysis of plastics with HZSM-5.^30,61^ The low methane yield is a direct consequence of the above-mentioned cracking mechanism of polyolefins on acidic catalysts. The formation of methane is intrinsically limited in catalytic cracking over HZSM-5, as C–C bond cleavage predominantly follows carbenium-ion-mediated β-scission pathways, the selectivity of which is governed by carbocation stability.^7^ In this context, primary carbenium species—such as the methyl carbenium ion (CH_3_^+^)—are highly unstable and therefore unlikely to participate as key intermediates compared to iso-carbenium ions.^14,62^ While minor contributions from free-radical methyl cracking cannot be fully ruled out, their impact is minimal relative to that of acid-catalyzed pathways, especially under the moderate operating temperatures considered in this study. Higher methane yield can be expected at higher operating temperature, in agreement with previous works.^63–66^

The effect of space-time on the compounds, according to their carbon atom number in the gasoline range (C_5_–C_11_), is shown in Figure 4. Hydrocarbons with five carbon atoms have olefinic and paraffinic nature, and their yield sharply decreases with increasing space-time because of intensified cracking toward gaseous compounds. More in detail, the C_5_ yield had a maximum of 3.91 wt % at the smaller space-time and decreased to 1.59 wt % at τ = 20 g_catalyst_ min g_plastic_^–1^. A significant share of compounds with 6, 7, and 8 carbon atoms is constituted by the aforementioned monoaromatics (BTX fraction), whose production is promoted by higher space-times. Although the nonaromatic fraction of the C_6_–C_8_ compounds decreases with increasing space-time due to enhanced cracking, BTX production is simultaneously favored by secondary condensation and oligomerization reactions. Finally, the C_9_–C_11_ fraction is almost insignificant (cracked to lighter compounds), with a decreasing trend with space-time.

**Figure 4:**
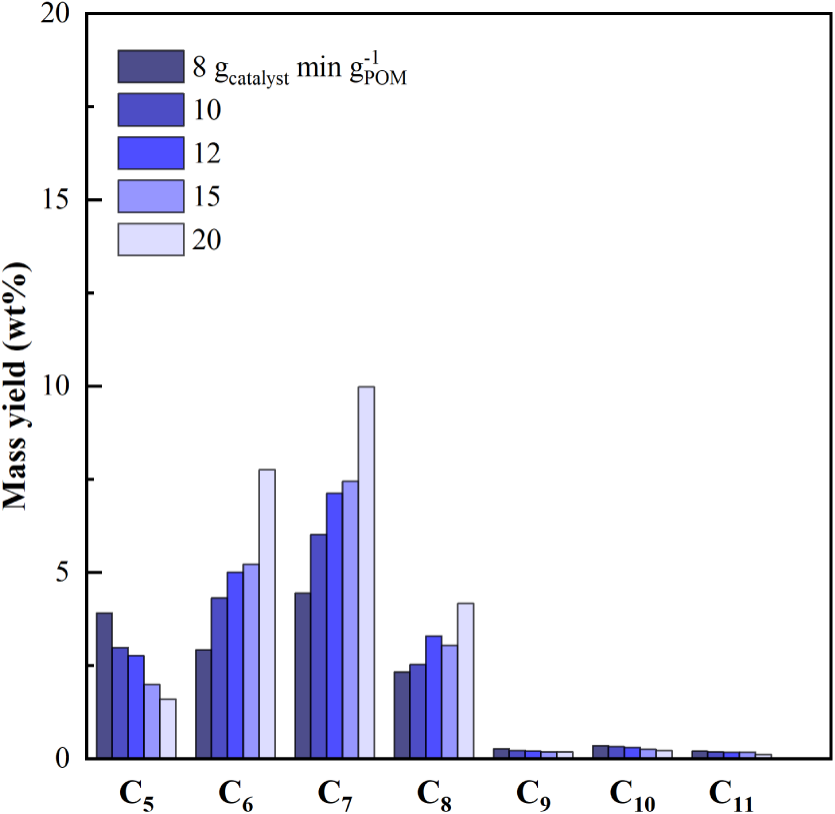
Effect of space-time on the gasoline fraction.

### 3.2 Evolution of Products with TOS: Effect of Operating Conditions on Product Distribution

Figure 5 shows the evolution of product fractions with TOS under different operating conditions of space-time (10 and 15 g_catalyst_ min g_plastic_^–1^) and temperature (450 and 500 °C). The experiments were conducted at a fixed pyrolysis temperature of 500 °C, while the operating parameters of the cracking reactor were varied. The adopted operating conditions are reported as follows:

1. *T*_cracking_ = 450 °C and τ = 10 g_catalyst_ min g_plastic_^–1^ (condition S1)
2. *T*_cracking_ = 500 °C and τ =10 g_catalyst_ min g_plastic_^–1^ (condition S2)
3. *T*_cracking_ = 500 °C and τ = 15 g_catalyst_ min g_plastic_^–1^ (condition S3)

**Figure 5:**
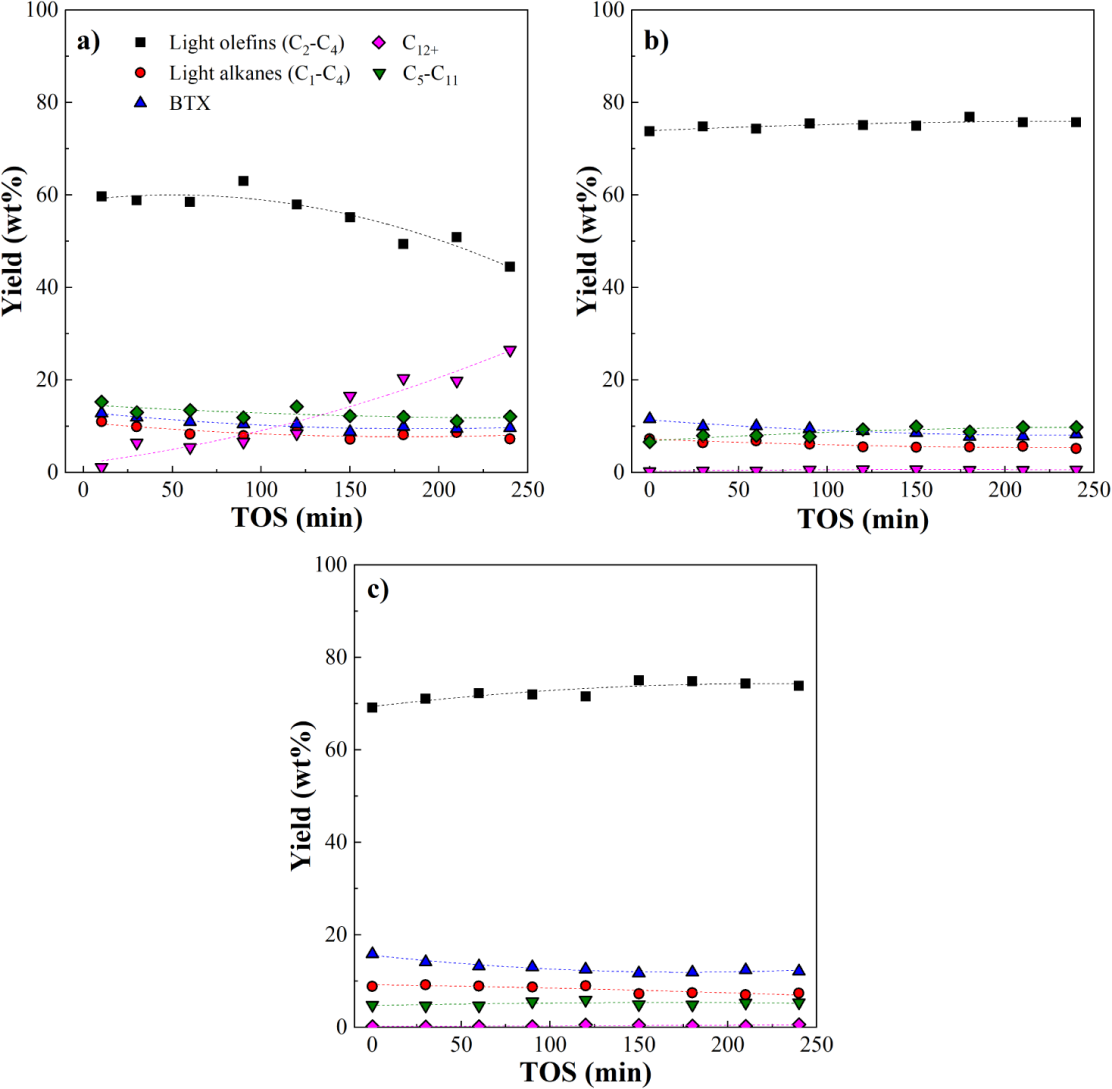
Effect of the operating conditions on product yields over time. Conditions: τ = 10 g_catalyst_ min g _plastic_^–1^ and 450 °C (a), τ = 10 g_catalyst_ min g _plastic_^–1^ and 500 °C (b), τ = 15 g_catalyst_ min g _plastic_^–1^ and 500 °C (c).

At 500 °C, the catalyst exhibited good stability over 240 min of operation under both space-time conditions, as indicated by the nearly constant light-olefin yield (Figure 5b and c). A slight decrease in BTX yield was observed over time, consistent with the trend reported by Elordi and coworkers.^18^ This behavior is associated with the high resistance of ZSM-5 to deactivation, attributed to its unique shape-selective pore structure, which sterically hinders the formation of polyaromatic coke precursors.^2,25,67–69^ A very slight deactivation of the catalyst was observed in the early stages of the reaction and was attributed to the coverage of strong acid sites by coke deposits, as also reported by Elordi and coworkers.^18^ At 450 °C (Figure 5a), the light olefin yield was substantially reduced compared to the trend observed at 500 °C, decreasing from 59.7 wt % at zero time on stream to 44.5 wt % after 4 h of continuous operation. It is to note that these results may be not only associated with the faster deactivation rate but also with the kinetic limitations operating at lower temperature. Across all experimental conditions, light alkane yield decreases with time on stream at a similar rate.

A monotonic decrease in BTX yield with TOS was observed under all experimental conditions, suggesting the deactivation of strong acid sites of the catalyst, which attenuates condensation reactions to yield aromatic hydrocarbons.^70^ Higher space-time led to increased BTX yields, in agreement with the results obtained for the fresh catalyst. More specifically, BTX decreased by 23.6% after 4 h with a space-time of 15 g_catalyst_ min g_plastic_^–1^ and by 28.1% at τ = 10 g_catalyst_ min g_plastic_^–1^. Individual BTX components (benzene, toluene, and xylenes) increased with space-time at 500 °C. Increasing temperature from 450 to 500 °C slightly increased benzene yield (from 1.70 to 2.05% at TOS = 0 min), decreased xylenes (from 5.78 to 4.17%), and had minimal effect on toluene (5.37% vs 5.36%). As a result of coke-induced catalyst deactivation, cracking of heavier hydrocarbon is weakened over time, resulting in an increase of C_5_–C_11_ and C_12+_ hydrocarbons. This is particularly evident at 450 °C.

In conclusion, at 500 °C, the process produced a gaseous stream exceeding 80 wt % in light hydrocarbons (C_1_–C_4_) over 4 h of continuous operation, with minimal catalyst deactivation. Less favorable conditions (450 °C and space-time of 10 g_catalyst_ min g_plastic_^–1^) resulted in a remarkable reduction of cracking activity throughout the continuous reaction. Therefore, a substantial reduction in light olefins yield and a high fraction of liquid hydrocarbons (∼48 wt %) was obtained after 4 h. A similar deactivation behavior was reported in previous literature in the cracking of waste plastics on HZSM-5 zeolites.^50^

The time evolution of individual compounds in the gas phase (H_2_ and C_1_–C_4_ compounds) is illustrated in Figure 6. The yields are substantially stable at 500 °C for both space-time conditions, reflecting high stability in the gas composition. Propylene, ethylene, and butenes consistently represented the most abundant compounds in the gas phase. At 500 °C, reducing the space-time from 15 to 10 min resulted in more butene and less ethylene. Yields of individual light alkanes also decrease with TOS, but the effect is less evident compared to light olefins, especially at 450 °C.

**Figure 6:**
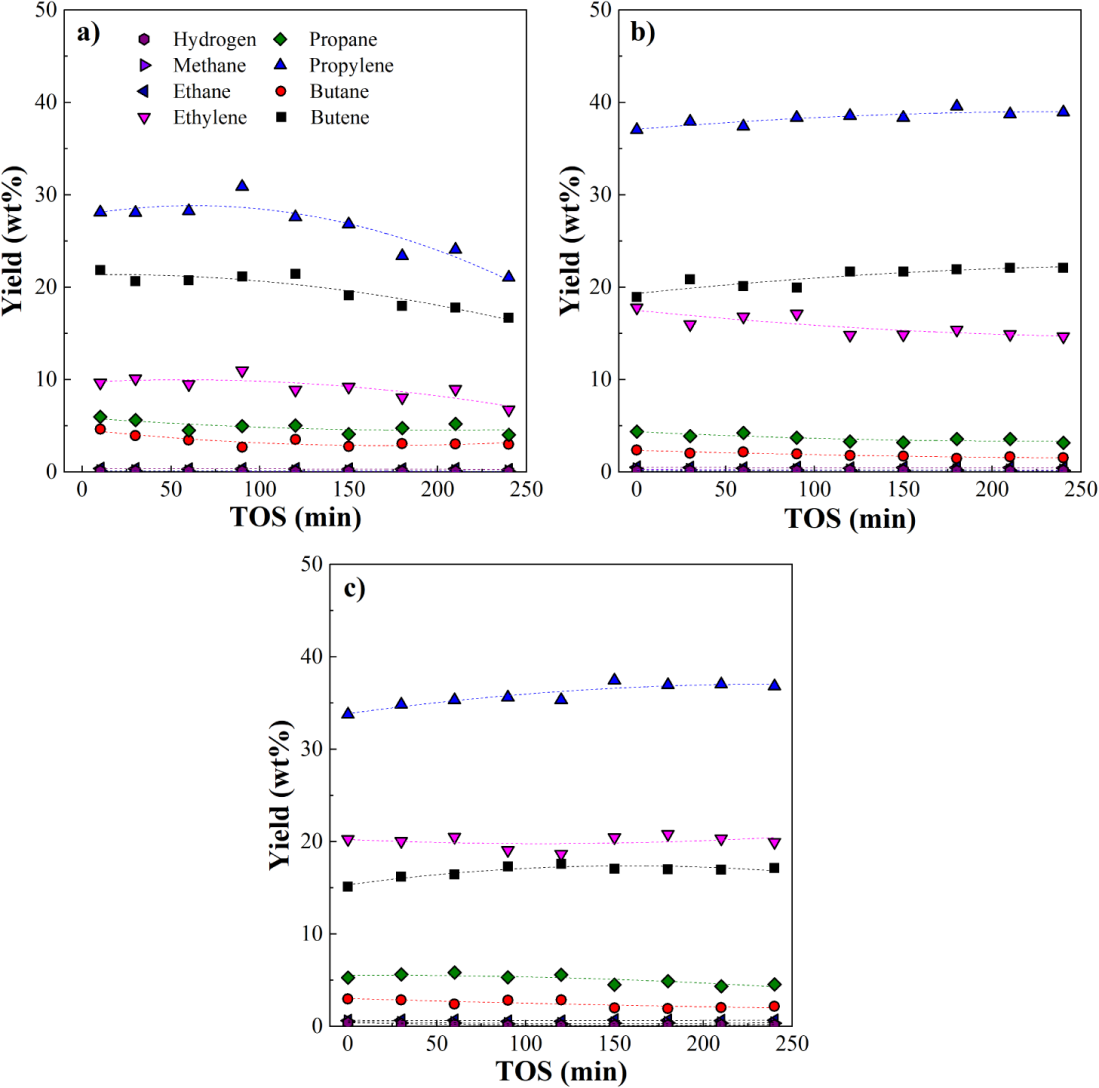
Individual gas-phase compound evolution with TOS. Conditions: τ = 10 g_catalyst_ min g _plastic_^–1^ and 450 °C (a), τ = 10 g_catalyst_ min g _plastic_^–1^ and 500 °C (b), τ = 15 g_catalyst_ min g _plastic_^–1^ and 500 °C (c).

### 3.3 Assessment of Catalyst Deactivation

In order to progress in the understanding of the catalyst deactivation process in polyolefin-derived volatile cracking, the properties of the spent catalyst have been investigated by characterizing the first and second bed sections, catalytic bed 1 (CB1) and catalytic bed 2 (CB2), respectively, at different reaction times. Thus, the physical properties and temperature-programmed oxidation (TPO) were analyzed to quantify morphological degradation and the amount and nature of coke, respectively.

BET surface area, micropore volume, and mesopore volume of spent catalysts collected at different TOS decreased progressively with increasing time on stream (Figure 7), with a sharp drop within the first 2 h followed by stabilization after ∼4 h, consistent with early-stage coke-induced deactivation of strong acid sites. At 500 °C and with the highest space-time (15 g_catalyst_ min g_plastic_^–1^), the BET surface areas of the first (CB1) and second section (CB2) of the catalytic bed decreased by 7.3% and 9.8% after 4 h, respectively. Corresponding reductions in micropore and mesopore volumes were 6.3% and 7.3% for CB1 and 11.3% and 7.3% for CB2. Reducing space-time amplified the difference in deactivation between the two portions of the bed. After 4 h, the BET surface area of CB1 decreased by 13.9%, while the second only declined by 7.9%. CB1 also shows greater deterioration compared to the experiment at higher space-time, consistent with the highest concentration of coke precursors in the reaction medium. At 450 °C, the catalyst suffered the strongest deactivation: the BET surface area decreased by 18.1% for CB1 and by 12.1% for CB2. As expected, CB1 experienced higher surface loss, because it directly receives high-molecular-weight volatiles coming from the spouted-bed reactor. Overall, deterioration severity followed the order S3 < S2 < S1, i.e., increasing with reducing temperature and space-time, consistent with coke-driven blockage of micropores and preferential loss of strong acid sites. These trends show that degradation is more severe compared to previous reports for HDPE and PP pyrolysis with homemade catalysts (with bentonite and alumina), which showed slower deactivation even after extended in situ and ex situ operation.^69–71^

**Figure 7:**
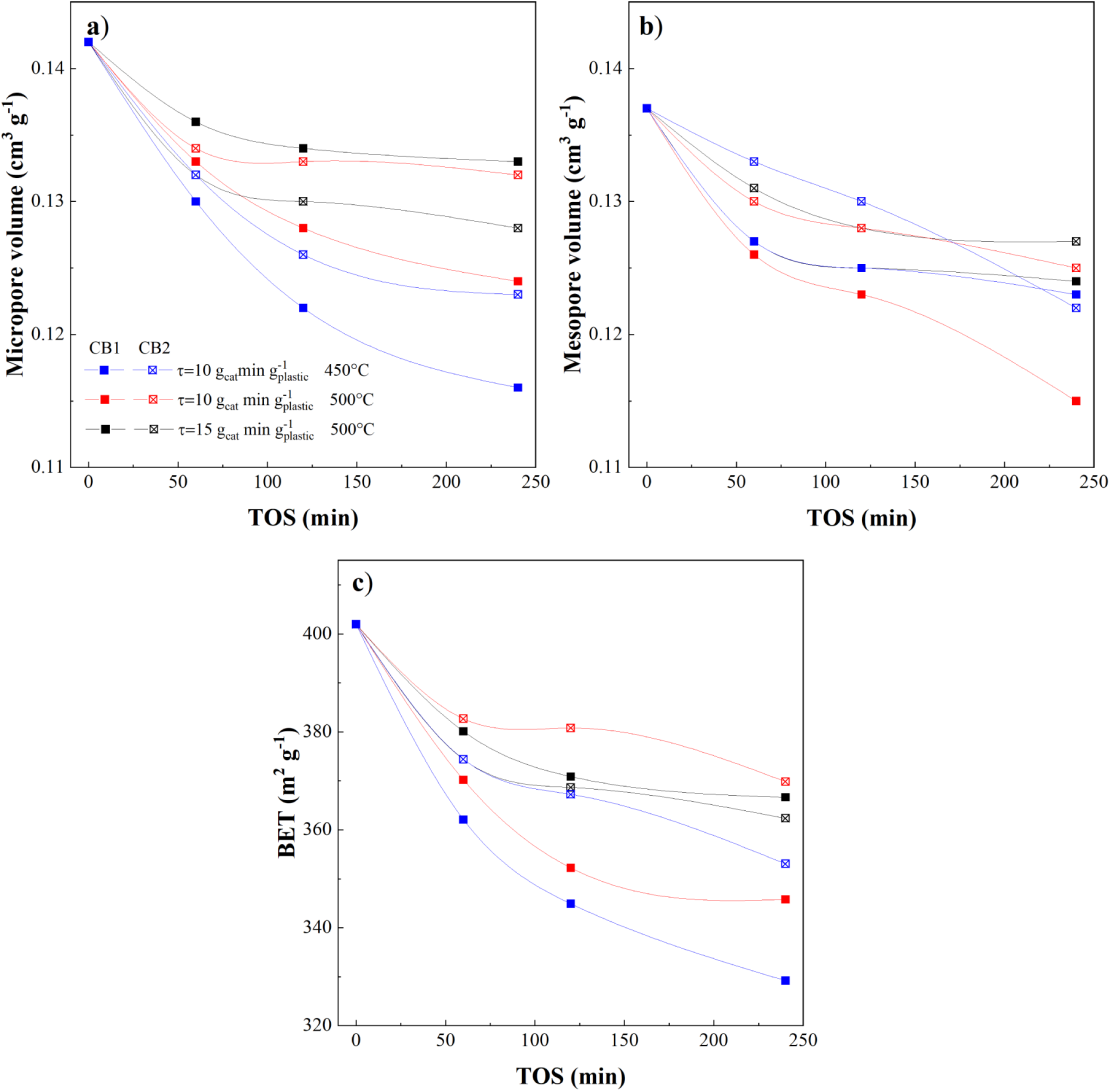
Effect of operating conditions on catalyst physical properties: a) micropore volume, b) mesopore volume, and c) BET surface area.

Coke deposition on spent catalyst samples collected at different reaction times was quantified by temperature-programmed oxidation (TPO), following the procedure described earlier. From the integration of DTG curves, it is possible to determine the amount of coke deposited on the catalyst, while the shape and combustion temperature of the peaks are related to the nature of the coke deposits.

Figure 8 shows the amount of coke deposited on the surface of the catalyst as a function of time on stream (TOS) under the three operating conditions examined. As observed in Figure 8, the coke formation rate is faster at the start of the reaction, and then, it attenuates for higher TOS values. The same qualitative effect has been previously reported in the plastic catalytic cracking on zeolites.^27,70^ Results revealed that coke content after 240 min of reaction increased when (i) space-time is reduced from 15 to 10 min; (ii) temperature is reduced from 500 to 450 °C, with mean values of 1.51%, 1.99%, and 2.26% in conditions S3, S2, and S1, respectively, in agreement with the trend observed for surface area and porosity. After 4 h of continuous operation, the coke accumulation remained low relative to the total catalyst mass. Approximately 200 mg of coke was produced per 240 g of plastic fed to the first reactor, corresponding to an overall coke yield below 0.1 wt %. The maximum coke content (2.82 wt %) was observed in the first catalytic bed at 450 °C and τ = 10 g_catalyst_ min g_plastic_, which is lower than the necessary coke deposition for complete micropore blockage.^72^ This can be related to the higher concentration of waxes, presumably the main precursors of coke, in the reaction medium at lower reaction temperatures. At 500 °C, the coke yield stabilized with increasing TOS, whereas at 450 °C it continued to rise after 4 h. The coke content in CB1 (in direct contact with pyrolysis waxes) is higher at low temperatures for the same space-time. Conversely, the coke content in CB2 is higher at 500 °C than at 450 °C. This highlights how different types of precursors (waxes, olefins, and aromatics) result in different distributions of coke on the catalyst. The composition of the gas phase changes along the axial position in the catalyst bed, from heavy waxes at the beginning to olefins and aromatic precursors in later sections.^70^

**Figure 8:**
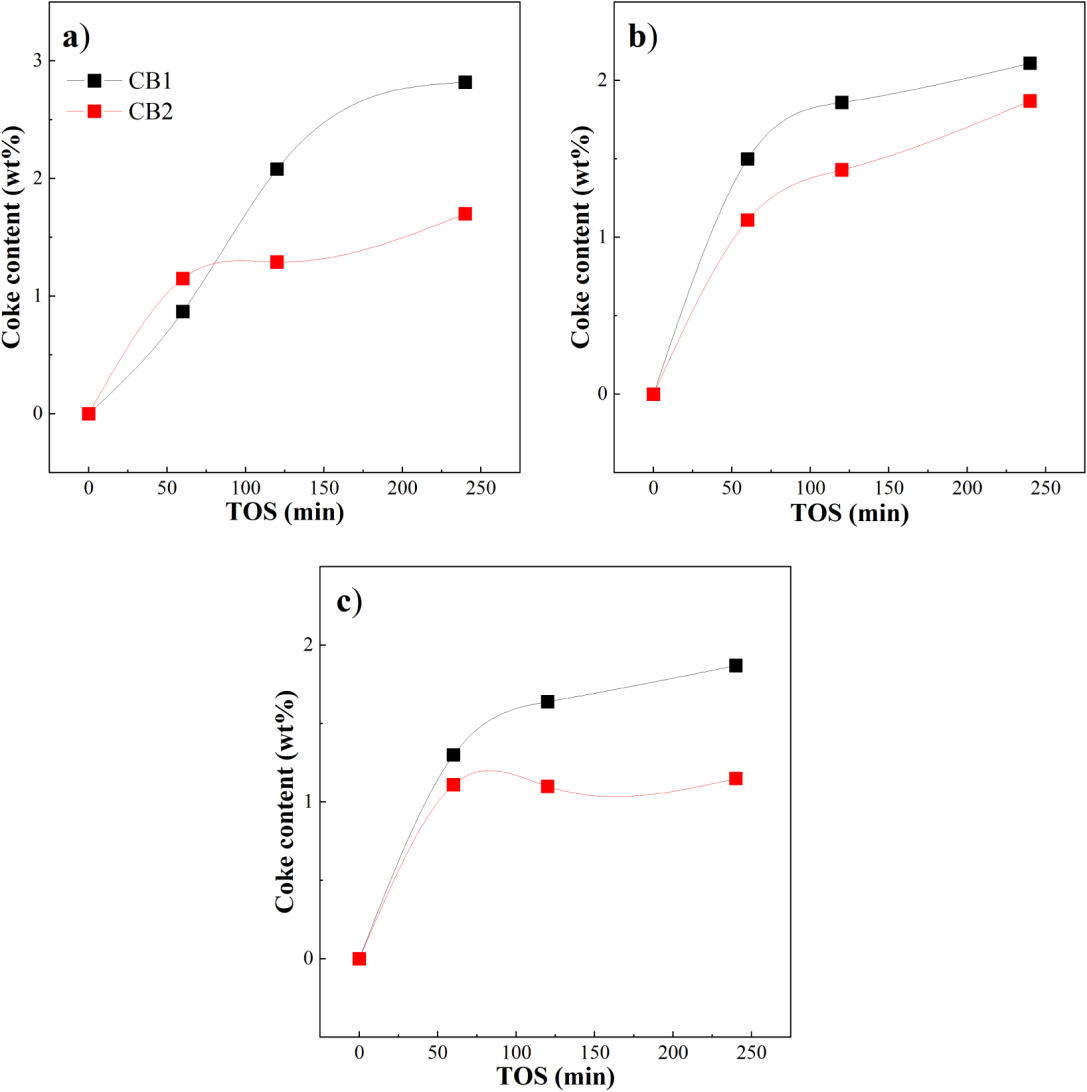
Amount of coke deposited on catalyst as a function of TOS. Conditions: τ = 10 g_catalyst_ min g _plastic_^–1^ and 450 °C (a), τ = 10 g_catalyst_ min g _plastic_^–1^ and 500 °C (b), τ = 15 g_catalyst_ min g _plastic_^–1^ and 500 °C (c).

Across all operating conditions—independent of space-time (10 or 15 g_catalyst_ min g_plastic_^–1^) and temperature (450 or 500 °C)—CB1 consistently accumulated more coke than CB2. This behavior, also reported in the literature,^48^ arises from CB1’s direct exposure to primary pyrolysis waxes, which are well-known coke precursors.^70^ The second bed, partially shielded from heavy volatiles, still contacts lighter coke precursors such as olefins and aromatics, which also contribute to coke formation.^46,73–76^ Compared to other catalysts like HY and Hβ, coke deposition is partially hindered by the unique pore structure of HZSM-5,^67,77,78^ which enhances stability by enabling coke precursor migration toward external surfaces, where they can be removed by the nitrogen flow.^21,69^ Its shape selectivity and acidity further promote monomolecular cracking over bimolecular pathways responsible for oligomerization and condensation leading to coke.^25^

Compared to the homemade catalyst agglomerated with alumina and bentonite,^22,30,48^ the commercial HZSM-5 employed here experienced higher coke deposition, consistent with its higher share of strong acidic sites and higher overall acidity.

Figure 9 shows the TPO profiles of spent catalysts as functions of temperature and time on stream (TOS) under the operating conditions investigated. For TOS ≥ 2 h, in the DTG, it is possible to distinguish two distinct peaks, with a maximum at ∼450 °C and ∼565 °C, respectively. This bimodal pattern is consistent with previous observations for HZSM-5 ^69^ and represents the sequential oxidation of less-structured and more highly condensed coke fractions.

**Figure 9:**
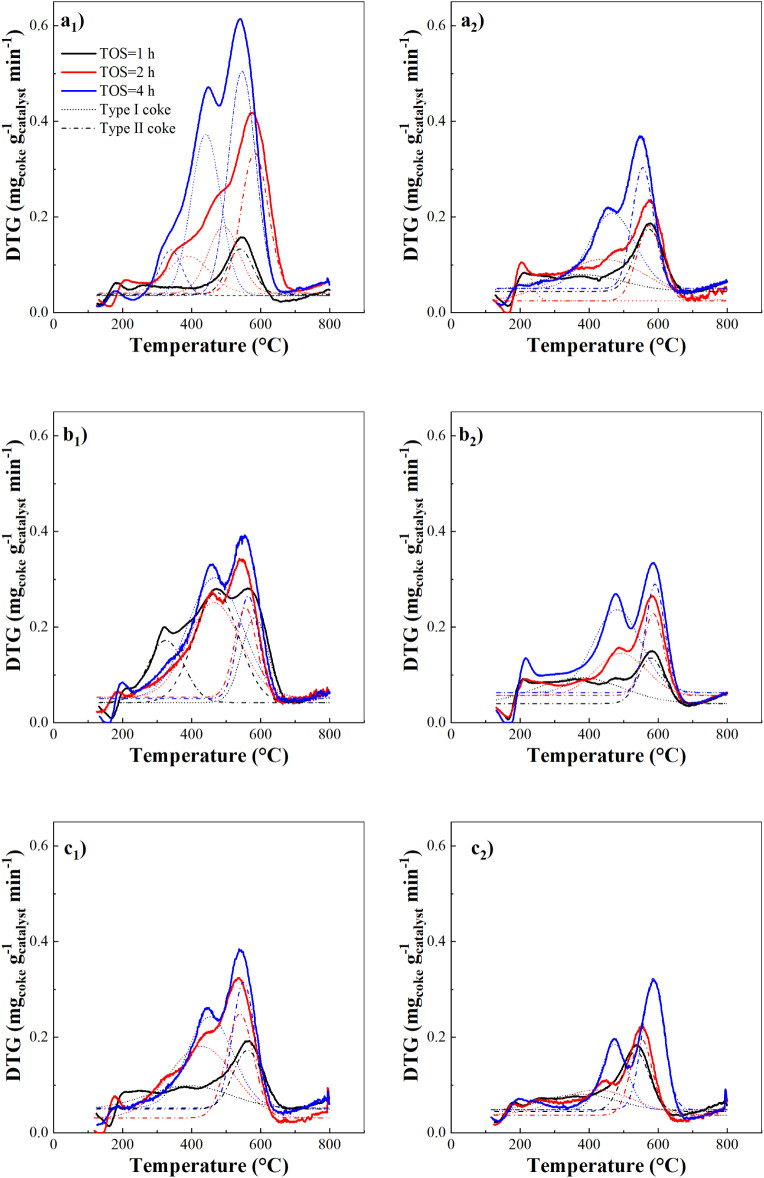
DTG profiles of the spent catalyst samples. Conditions: τ = 10 g_catalyst_ min g _plastic_^–1^ and 450 °C (a), τ = 10 g_catalyst_ min g _plastic_^–1^ and 500 °C (b), τ = 15 g_catalyst_ min g _plastic_^–1^ and 500 °C (c). Left column refers to results for the first catalytic bed (CB1) and right to the second one (CB2).

DTG curves were deconvoluted by multipeak Gaussian fitting (four-parameter Levenberg–Marquardt algorithm) to quantify the relative contributions of the two characteristic coke families (type I and type II), following established procedures.^69–71^ These coke types differ in chemical nature (degree of polyaromatic condensation), H/C ratio, and location within the zeolite structure.

Type I coke is mainly situated in meso- and macropores (high O_2_ availability), has a higher H/C ratio, and is mainly derived from oligomerization, condensation, and alkylation of pyrolysis volatiles, associated with the retention of waxes and heavy compounds in mesoporous regions.^25,48,79^ Conversely, type II coke burns at higher temperature, is composed of highly condensed polyaromatic residue, and is typically confined in micropores, where it has the time to evolve toward more structured compounds. At higher temperatures, cyclization, cracking, and hydrogen transfer inside micropores promote the formation of type II coke, composed of dense, stable polyaromatic species.^25^ Type I coke is partially reversible and can be reconverted to noncondensed intermediates under suitable conditions,^79^ while type II coke is highly stable and irreversible.^46^ Coke content increased with time on stream (TOS) for both sections of the catalytic bed, and the peak associated with type II coke is always higher compared with type I, in agreement with the sharp decrease in micropore volume. For instance, under condition S1 (240 min on stream), coke I amounted to 1.37% in CB1 and 0.97% in CB2, while coke II reached 1.45% and 0.73%, respectively. At the same space-time but at 500 °C (condition S2), coke I was 1.59% in CB1 and 1.29% in CB2, whereas coke II was 0.52% in CB1 and 0.64% in CB2. At higher space-time (S3), coke I reached 1.12% in CB1 and 0.43% in CB2, while coke II was 0.75% and 0.71% in CB1 and CB2, respectively, after 240 min of continuous operation.

In summary, the deactivation study of HZSM-5 during in-line catalytic cracking of polyolefin volatiles identifies temperature as the key factor controlling catalyst stability and performance. Operating at 500 °C is thus necessary to promote complete cracking of coke I precursors, i.e, waxes, and, at the same time, attenuate the formation of coke II, preserving catalyst activity and stability.

## 4 Conclusions

This study shows that different polyolefins can be efficiently valorized through a two-step pyrolysis process to generate valuable products, most notably light olefins, under moderate space-time and temperature conditions. The spouted-bed reactor consistently provided a high-quality volatile stream enriched in waxes, well-suited for catalytic upgrading on HZSM-5. The catalyst showed good stability and high selectivity toward light olefins, with a maximum of 77 wt % at a space-time of 10 g_catalyst_ min g_plastic_^–1^. Increasing space-time beyond this point led to a reduction in light olefin yield, while BTX production increased monotonically with space-time, reflecting their formation through secondary reactions of olefinic intermediates. The catalyst maintained satisfactory activity and stability over 240 min of continuous operation, with limited deactivation and coke deposition (<3 wt %). It was shown that a cracking temperature of 500 °C is suitable for sustained production of light olefins from plastic waste, while lower temperature results in higher coke deposition and faster deactivation. Future work will address the use of real postconsumer waste polyolefins to account for compositional variability and the presence of contaminants.

## Data Availability

Data will be made available upon request.
